# A phase Ib trial of combined PKC and MEK inhibition with sotrastaurin and binimetinib in patients with metastatic uveal melanoma

**DOI:** 10.3389/fonc.2022.975642

**Published:** 2023-06-09

**Authors:** Sebastian Bauer, James Larkin, F. Stephen Hodi, Frank Stephen, Ellen H. W. Kapiteijn, Gary K. Schwartz, Emilano Calvo, Padmaja Yerramilli-Rao, Sophie Piperno-Neumann, Richard D. Carvajal

**Affiliations:** ^1^ Department of Medical Oncology, Sarcoma Center, West German Cancer Center, University Duisburg-Essen, Medical School, Essen, Germany; ^2^ Department of Medical Oncology and Hematology, The Royal Marsden Hospital, London, United Kingdom; ^3^ Melanoma Center and Center for Immuno-Oncology, Dana−Farber Cancer Institute, Boston, MD, United States; ^4^ Hebrew University Hadassah Medical School, The Sharett Institute of Oncology, Jerusalem, Israel; ^5^ Jacob Schachter, Sheba Medical Center at Tel Hashomer, Tel-Aviv University Medical School, Tel Aviv, Israel; ^6^ Department of Medical Oncology, Leiden University Medical Centre, Leiden, Netherlands; ^7^ Division of Hematology and Oncology, Columbia University Irving Medical Center, New York, NY, United States; ^8^ Early Phase Clinical Drug Development in Oncology, START Madrid-CIOCC, Centro Integral Oncológico Clara Campa, Madrid, Spain; ^9^ Translational Clinical Oncology, Novartis Institutes for BioMedical Research, Cambridge, MA, United States; ^10^ Department of Medical Oncology, Institut Curie, Paris, France

**Keywords:** uveal melanoma, GNAQ, GNA11, binimetinib, MEK, sotrastaurin, PKC

## Abstract

**Background:**

Uveal melanoma is a disease characterized by constitutive activation of the G alpha pathway and downstream signaling of protein kinase C (PKC) and the mitogen-activated protein kinase (MAPK) pathway. While limited clinical activity has been observed in patients with metastatic disease with inhibition of PKC or MEK alone, preclinical data has demonstrated synergistic antitumor effects with concurrent inhibition of PKC and MEK.

**Method:**

We conducted a phase Ib study of the PKC inhibitor sotrastaurin in combination with the MEK inhibitor binimetinib in patients with metastatic uveal melanoma using a Bayesian logistic regression model guided by the escalation with overdose control principle (NCT01801358). Serial blood samples and paired tumor samples were collected for pharmacokinetic (PK) and pharmacodynamic analysis.

**Results:**

Thirty-eight patients were treated across six dose levels. Eleven patients experienced DLTs across the five highest dose levels tested, most commonly including vomiting (n=3), diarrhea (n=3), nausea (n=2), fatigue (n=2) and rash (n=2). Common treatment related adverse events included diarrhea (94.7%), nausea (78.9%), vomiting (71.1%), fatigue (52.6%), rash (39.5%), and elevated blood creating phosphokinase (36.8%). Two dose combinations satisfying criteria for the maximum tolerated dose (MTD) were identified: (1) sotrastaurin 300 mg and binimetinib 30 mg; and, (2) sotrastaurin 200 mg and binimetinib 45 mg. Exposure to both drugs in combination was consistent with single-agent data for either drug, indicating no PK interaction between sotrastaurin and binimetinib. Stable disease was observed in 60.5% of patients treated. No patient achieved a radiographic response per RECIST v1.1.

**Conclusions:**

Concurrent administration of sotrastaurin and binimetinib is feasible but associated with substantial gastrointestinal toxicity. Given the limited clinical activity achieved with this regimen, accrual to the phase II portion of the trial was not initiated.

## 1 Introduction

Uveal melanoma is a malignant neoplasm of the eye which is biologically distinct from melanoma of the skin. Although representing only 3% to 5% of all melanomas, it is the most common primary intraocular malignant tumor in adults (1). Arising in the pigmented portions of the eye including the choroid, ciliary body or iris, uveal melanoma has a population incidence of approximately five cases per million in the US (2). Up to 50% of patients with uveal melanoma develop metastatic disease within 15 years of initial diagnosis (3). Frequent sites of metastasis include the liver, lungs, bone, and skin, and, less commonly, the brain and lymph nodes. The historical median survival for patients with metastatic disease is approximately 12 months, with a one-year survival of approximately 43% (4, 5); however, for previously untreated patients who are HLA-A*0201 positive, tebentafusp has recently been demonstrated to improve overall survival when compared to best alternative care, with a hazard ratio for death of 0.51, representing a reduction in the risk of death of 49% with tebentafusp (6, 7).

Somatic mutations leading to constitutive activation of the G alpha pathway arise early in the development of uveal melanoma and are characteristic of this disease. Mutations in either *GNAQ* or *GNA11*, genes that encode G protein alpha subunits of heterotrimeric G protein-coupled receptor (GPCR) complexes, have been identified in 96% of patients with metastatic uveal melanoma (8, 9). Other common alterations include PLCB4 and CYSLTR2, as well as EIFA1X, SF3B1 and BAP1. The later three have been associated with prognosis and SF3B1 and BAP1 are being explored as predictive biomarkers of response to various targeted and epigenetic therapeutic approaches (10–12). Protein kinase C (PKC) and the mitogen-activated protein kinase (MAPK) pathway, both of which are downstream of the G alpha pathway, have been implicated in the pathogenesis of uveal melanoma. Modest activity has been observed in clinical trials targeting PKC or MEK alone in patients with advanced disease (13, 14). Given the improved efficacy achieved with concurrent inhibition of both BRAF and MEK, when compared with BRAF inhibition alone, in melanomas harboring BRAF mutations, we hypothesized that concurrent inhibition of PKC and MEK in uveal melanomas driven by activation of the G alpha signaling pathway may similarly result in improved outcomes.

Binimetinib (MEK162) is a potent and selective oral, adenosine triphosphate noncompetitive, small-molecule inhibitor of MEK1/2 approved for the treatment of BRAF mutant melanoma when given in combination with the BRAF inhibitor encorafenib (15, 16). Sotrastaurin (AEB071) is a potent, oral, selective inhibitor of the classical (α, β) and novel (δ, ϵ, η, θ) PKC isoforms (17). Synergistic antitumor effects were observed with the combination of binimetinib and sotrastaurin in preclinical models of uveal melanoma harboring *GNAQ* or *GNA11* mutations (18).

We therefore conducted this phase Ib study of the combination of sotrastaurin and binimetinib in patients with metastatic uveal melanoma, with the primary objectives of identifying the recommended phase II dose (RP2D), obtaining preliminary data of the efficacy of this combination, and informing the future development of this regimen.

## 2 Materials and methods

### 2.1 Study patients

Patients 18 years or older with biopsy confirmed metastatic uveal melanoma measurable using Response Evaluation Criteria in Solid Tumors (RECIST) v1.1 and a World Health Organization (WHO) performance status (PS) ≤ 1 were considered eligible. Patients may have been previously untreated or received any number of lines of therapy prior to study entry; however, patients could not have received prior therapy with a PKC or MEK inhibitor. The clinical trial was approved by the relevant Institution Review Boards at participating centers and was conducted in accordance with the Declaration of Helsinki and International Conference for Harmonization of Good Clinical Practice Guidelines. All patients provided written informed consent for participating in the study.

### 2.2 Study design and treatment

In this multicenter, open-label, phase Ib study, eligible patients received sotrastaurin and binimetinib, each administered orally twice daily (b.i.d.) without food. Six dose levels were evaluated: dose level 1 - sotrastaurin 150 mg b.i.d. and binimetinib 45 mg b.i.d.; dose level 2 - sotrastaurin 200 mg b.i.d. and binimetinib 45 mg b.i.d.; dose level 3 - sotrastaurin 300 mg b.i.d. and binimetinib 30 mg b.i.d.; dose level 4 - sotrastaurin 300 mg b.i.d. and binimetinib 45 mg b.i.d.; dose level 5 - sotrastaurin 350 mg b.i.d. and binimetinib 30 mg b.i.d.; and, dose level 6 - sotrastaurin 400 mg b.i.d. and binimetinib 30 mg b.i.d. Of note, accrual was initiated at dose level 6 and progressively de-escalated based upon tolerability. Treatment cycles were 28 days, given without interruption (continuous cycles). Patients continued treatment as long as clinical benefit was seen and no limiting adverse toxicity was observed.

### 2.3 Safety and response evaluation

Treated patients were assessed for toxicity by physical examinations on a weekly basis during cycle 1 and on day 1 of each subsequent cycle, with laboratory assessments and ECGs performed on cycle 1 day 1, cycle 1 day 15, and on day 1 of each subsequent cycle. Patients underwent imaging studies for response evaluation during screening and after every 2 cycles of treatment. All patients who received at least one dose of sotrastaurin or binimetinib and had at least one valid post-baseline safety assessment were considered evaluable for toxicity. Patients were considered eligible for dose limiting toxicity assessment (DLT) if they received at least 21 of the 28 planned daily doses of both sotrastaurin and binimetinib in the first 28 days of the dosing regimen. Patients who did not experience a DLT during the first cycle were considered to have sufficient safety evaluations if they had been observed for ≥ 28 days following the first dose and were considered to have enough safety data to conclude that a DLT had not occurred.

### 2.4 Pharmacokinetic studies

Blood samples for pharmacokinetic (PK) analysis of sotrastaurin and binimetinib were collected and evaluated for all patients participating in phase Ib of the study. Samples were collected on days 1, 8, 15 and 22 of cycle 1, and pre-dose on day 1 of cycles 2 through 6. Plasma concentrations were measured using a validated liquid chromatography-tandem mass spectrometry assay. The lower limit of quantification (LLOQ) was 3.0 ng/mL for sotrastaurin and its metabolite AEE800. The LLOQ was 1.0 ng/mL for binimetinib and its metabolite AR00426032. Concentrations below the LLOQ were treated as zero in summary statistics.

### 2.5 Pharmacodynamic studies

Tumor samples were collected at baseline and after 2 weeks of therapy using either CT-guided or ultrasound-guided biopsies in order to assess if sotrastaurin and/or binimetinib inhibits the PKC and/or MAPK pathways in tumors. The expression levels of MARCKS, pMARCKS, ERK, pERK and the relative value of pMARCKS over total MARCKS and pERK over total ERK were evaluated using electrochemiluminescent assays on the Meso Scale Discovery Platform using whole cell lysate kits (MSD-ECL) and performed by BioAgilytix, Boston, MA (19).

### 2.6 Statistical considerations

A Bayesian logistic regression model (BLRM) guided by the escalation with overdose control (EWOC) principle, which is a well-established, appropriate method to estimate the MTD and/or RP2D in cancer patients (20), was utilized to estimate the MTD of the combination treatment. Dose recommendations were based on summaries of the posterior distribution of model parameters and the posterior distribution of DLT rates, including the mean, median, standard deviation, 95% credibility interval, and the probability that the true DLT rate for each dose combination lies in one of the following categories: [0%, 16%] under-dosing; [16%, 35%] targeted toxicity; or [35%, 100%] excessive toxicity. Following the principle of EWOC, after each cohort of patients the recommended dose combination was the one with the highest posterior probability of DLT in the target interval [16%, 35%] among the doses fulfilling the overdose criterion that there was less than 25% chance of excessive toxicity.

Assessment of preliminary efficacy was based on BOR as defined by the RECIST v1.1: progressive disease (PD), stable disease (SD), partial response (PR) and complete response (CR). Kaplan-Meier analysis was utilized to assess progression-free survival (PFS).

All primary PK parameters (area under the curve [AUC_0-8h_], maximum plasma concentration [C_max_], time point of maximum concentration [T_max_], and accumulation ratio [R_ACC_]) were calculated for binimetinib and sotrastaurin by dose level. The PK analyses used the actual dose received for each particular PK profile. Parameters relating to the PK profile (e.g., AUC and C_max_) were summarized for data collected on C1D1 and C1D15.

## 3 Results

### 3.1 Patient characteristics

A total of 38 patients were enrolled in the phase Ib part of this study in 16 sites in the United States and Europe (France, Germany, the Netherlands, Spain and the United Kingdom). Six patients were enrolled in each dose level, except for the dose level 6 (sotrastaurin 400 mg b.i.d. and binimetinib 30 mg b.i.d.) which included eight patients.

The median age of all patients was 57 years (**Table 1**). The majority (81.6%) of patients were Caucasian with a higher proportion of males (63.2%). The majority (84.2%) of patients had WHO PS scores of 0 at baseline. Liver metastases were reported in 35 (92.1%) patients, with 3 patients having extrahepatic metastases only.

**Table 1:** Baseline patient characteristics.

	Dose Level 1	Dose Level 2	Dose Level 3	Dose Level 4	Dose Level 5	Dose Level 6	All patients(n = 38)
	AEB071 150mg bid and MEK162 45mg bid (n = 6)	AEB071 200mg bid and MEK162 45mg bid (n = 6)	AEB071 300mg bid and MEK162 30mg bid (n = 6)	AEB071 300mg bid and MEK162 45mg bid (n = 6)	AEB071 350mg bid and MEK162 30mg bid (n = 6)	AEB071 400mg bid and MEK162 30mg bid (n = 8)	
Median age in years (range)	49.5 (25–71)	59.5 (39–68)	53.0 (38–68)	60.5 (47–70)	59.5 (37–66)	61.5 (42–73)	57.0 (25–73)
Sex, n (%)							
Male	3 (50.0)	4 (66.7)	3 (50.0)	4 (66.7)	4 (66.7)	6 (75.0)	24 (63.2)
Female	3 (50.0)	2 (33.3)	3 (50.0)	2 (33.3)	2 (33.3)	2 (25.0)	14 (36.8)
Baseline WHO PS, n (%)							
0	3 (50.0)	6 (100)	5 (83.3)	5 (83.3)	5 (83.3)	8 (100)	32 (84.2)
1	3 (50.0)	0	1 (16.7)	1 (16.7)	1 (16.7)	0	6 (15.8)
Median number of prior systemic therapies in metastatic setting (range)	1 (0-2)	1 (0-2)	0 (0-2)	1 (0-2)	2 (0-2)	0 (0-1)	1 (0-1)
Patients treated with ipilimumab	2	0	1	2	5	0	10
Patients treated with anti-PD1	0	0	0	0	1	0	1
Patient treated with targeted therapy	0	2	0	0	1	0	3
Extent of Disease							
Hepatic Only	1	3	1	1	2	0	8
Hepatic and Extrahepatic	5	2	5	5	4	6	27
Extrahepatic Only	0	1	0	0	0	2	3
Median number of organ sites involved (range)	2 (1-6)	2 (1-3)	2 (1-8)	3 (1-5)	2 (1-5)	4 (2-8)	3 (1-8)

AEB071, sotrastaurin; bid, twice a day; MEK162, binimetinib; PS, performance status; WHO, World Health Organization.

### 3.2 Safety and tolerability

All patients were considered evaluable for safety and DLT determination. All patients experienced at least one AE regardless of relationship to study treatment during the study (**Table 2**). Gastrointestinal-related AEs were the most commonly reported toxicities, including diarrhea (97.4%), nausea (81.6%) and vomiting (78.9%). The most frequently reported grade 3 or 4 AEs were nausea, vomiting, and increased blood creatinine phosphokinase (CPK; 15.8%), as well as anemia (13.2%).

**Table 2:** Adverse events, regardless of attribution, reported in ≥ 15% of all patients.

	Dose Level 1		Dose Level 2		Dose Level 3		Dose Level 4		Dose Level 5		Dose Level 6		All patients(n = 38)	
	AEB071 150 mg bidandMEK162 45 mg bid(n = 6)		AEB071 200 mg bid andMEK162 45 mg bid(n = 6)		AEB071 300 mg bid andMEK162 30 mg bid(n = 6)		AEB071 300 mg bid andMEK162 45 mg bid(n = 6)		AEB071 350 mg bid andMEK162 30 mg bid(n = 6)		AEB071 400 mg bid andMEK162 30 mg bid(n = 8)			
	All grades: n (%)	Grade 3/4 n (%)	All grades: n (%)	Grade 3/4 n (%)	All grades: n (%)	Grade 3/4 n (%)	All grades: n (%)	Grade 3/4 n (%)	All grades: n (%)	Grade 3/4 n (%)	All grades: n (%)	Grade 3/4 n (%)	All grades: n (%)	Grade 3/4 n (%)
**Total**	**6 (100)**	**5 (83.3)**	**6 (100)**	**5 (83.3)**	**6 (100)**	**6 (100)**	**6 (100)**	**5 (83.3)**	**6 (100)**	**3 (50.0)**	**8 (100)**	**7 (87.5)**	**38 (100)**	**31 (81.6)**
Diarrhea	6 (100)	0	5 (83.3)	0	6 (100)	1 (16.7)	6 (100)	2 (33.3)	6 (100)	0	8 (100)	1 (12.5)	37 (97.4)	4 (10.5)
Nausea	6 (100)	1 (16.7)	3 (50.0)	1 (16.7)	4 (66.7)	0	6 (100)	2 (33.3)	4 (66.7)	0	8 (100)	2 (25.0)	31 (81.6)	6 (15.8)
Vomiting	5 (83.3)	0	3 (50.0)	1 (16.7)	5 (83.3)	0	5 (83.3)	2 (33.3)	5 (83.3)	1 (16.7)	7 (87.5)	2 (25.0)	30 (78.9)	6 (15.8)
Fatigue	3 (50.0)	0	3 (50.0)	0	4 (66.7)	2 (33.3)	4 (66.7)	2 (33.3)	3 (50.0)	0	5 (62.5)	0	22 (57.9)	4 (10.5)
Peripheral edema	2 (33.3)	0	2 (33.3)	1 (16.7)	4 (66.7)	0	4 (66.7)	1 (16.7)	2 (33.3)	0	3 (37.5)	0	17 (44.7)	2 (5.3)
Increased blood creatinine phosphokinase	4 (66.7)	2 (33.3)	5 (83.3)	2 (33.3)	1 (16.7)	0	2 (33.3)	0	3 (50.0)	1 (16.7)	2 (25.0)	1 (12.5)	17 (44.7)	6 (15.8)
Rash	5 (83.3)	2 (33.3)	2 (33.3)	0	2 (33.3)	0	3 (50.0)	1 (16.7)	2 (33.3)	0	1 (12.5)	0	15 (39.5)	3 (7.9)
Asthenia	3 (50.0)	1 (16.7)	2 (33.3)	0	2 (33.3)	1 (16.7)	2 (33.3)	0	0	0	3 (37.5)	1 (12.5)	12 (31.6)	3 (7.9)
Increased aspartate aminotransferase	1 (16.7)	0	4 (66.7)	1 (16.7)	1 (16.7)	0	3 (50.0)	2 (33.3)	3 (50.0)	0	0	0	12 (31.6)	3 (7.9)
Dermatitis acneiform	0	0	3 (50.0)	2 (33.3)	3 (50.0)	0	3 (50.0)	0	2 (33.3)	1 (16.7)	0	0	11 (28.9)	3 (7.9)
Constipation	2 (33.3)	0	2 (33.3)	0	1 (16.7)	0	0	0	1 (16.7)	0	4 (50.0)	0	10 (26.3)	0
Decreased appetite	1 (16.7)	0	2 (33.3)	0	1 (16.7)	0	2 (33.3)	1 (16.7)	1 (16.7)	0	3 (37.5)	0	10 (26.3)	1 (2.6)
Anemia	1 (16.7)	0	0	0	3 (50.0)	1 (16.7)	4 (66.7)	3 (50.0)	0	0	1 (12.5)	1 (12.5)	9 (23.7)	5 (13.2)
Dysgeusia	2 (33.3)	0	2 (33.3)	0	1 (16.7)	0	2 (33.3)	0	2 (33.3)	0	0	0	9 (23.7)	0
Chorioretinopathy	1 (16.7)	0	1 (16.7)	0	0	0	0	0	1 (16.7)	0	5 (62.5)	0	8 (21.1)	0
Decreased weight	0	0	0	0	2 (33.3)	0	3 (50.0)	0	1 (16.7)	0	2 (25.0)	0	8 (21.1)	0
Dyspnea	4 (66.7)	0	0	0	1 (16.7)	0	2 (33.3)	1 (16.7)	0	0	1 (12.5)	0	8 (21.1)	1 (2.6)
Pruritis	2 (33.3)	0	2 (33.3)	0	2 (33.3)	0	1 (16.7)	0	0	0	1 (12.5)	0	8 (21.1)	0
Retinal detachment	2 (33.3)	0	3 (50.0)	0	1 (16.7)	0	1 (16.7)	0	0	0	0	0	7 (18.4)	0
	All grades: n (%)	Grade 3/4 n (%)	All grades: n (%)	Grade 3/4 n (%)	All grades: n (%)	Grade 3/4 n (%)	All grades: n (%)	Grade 3/4 n (%)	All grades: n (%)	Grade 3/4 n (%)	All grades: n (%)	Grade 3/4 n (%)	All grades: n (%)	Grade 3/4 n (%)
Blurred vision	0	0	2 (33.3)	0	0	0	2 (33.3)	0	1 (16.7)	0	2 (25.0)	0	7 (18.4)	0
Abdominal pain	1 (16.7)	0	2 (33.3)	0	3 (50.0)	0	1 (16.7)	0	0	0	0	0	7 (18.4)	0
Rash pustular	0	0	0	0	0	0	0	0	4 (66.7)	1 (16.7)	2 (25.0)	0	6 (15.8)	1 (2.6)

In descending frequency of total grades (for ≥ 15% of all patients).

AEB071, sotrastaurin; bid, twice a day; MEK162, binimetinib.

Bold values summarizes the number of patients that were affected by any of the listed side effects.

Overall, 37 (97.4%) patients experienced a treatment related adverse event (TRAE), with 26 (68.4%) patients experiencing a grade 3 or 4 TRAE. The most frequently reported TRAEs included diarrhea (94.7%), nausea (78.9%), vomiting (71.1%), fatigue (52.6%), rash (39.5%), and increased blood creatine phosphokinase (36.8%). Three (7.9%) patients had a QTcF post-baseline increase > 60 ms: 1 patient each in dose level 2 (sotrastaurin 200 mg b.i.d. and binimetinib 45 mg b.i.d.), dose level 3 (sotrastaurin 300 mg b.i.d. and binimetinib 30 mg b.i.d.), and dose level 5 (sotrastaurin 350 mg b.i.d. and binimetinib 30 mg b.i.d.). Two (5.3%) patients had a new QTcF interval > 500 ms: 1 patient each in dose level 2 (sotrastaurin 200 mg b.i.d. and binimetinib 45 mg b.i.d.) and dose level 6 (sotrastaurin 400 mg b.i.d. and binimetinib 30 mg b.i.d.). No episodes of torsades de pointes were identified.

Thirty (78.9%) patients experienced at least one AE requiring dose adjustment or treatment interruption (**Table 3**), with similar frequency across all dose levels (66.7% to 87.5%). The most frequently (≥ 5 patients in all patients) reported adverse events requiring dose adjustment or study treatment interruption included: nausea (39.5%), vomiting (34.2%), diarrhea (18.4%), increased blood CPK (15.8%), chorioretinopathy (13.2%) and fatigue (13.2%). Dose reductions were more commonly applied to sotrastaurin (76.3%) than to binimetinib (65.8%). Dose interruptions of binimetinib and sotrastaurin were reported in all dose levels. The median number of dose delays per patient was 2, with a median cumulative dose delay duration of 14 days. Four (10.5%) patients experienced AEs leading to study treatment discontinuation.

**Table 3:** Dose limiting toxicities occurring during cycle 1.

	Dose Level 1	Dose Level 2	Dose Level 3	Dose Level 4	Dose Level 5	Dose Level 6	All patients (n = 38)
	AEB071 150mg bid and MEK162 45mg bid (n = 6)	AEB071 200mg bid and MEK162 45mg bid (n = 6)	AEB071 300mg bid and MEK162 30mg bid (n = 6)	AEB071 300mg bid and MEK162 45mg bid (n = 6)	AEB071 350mg bid and MEK162 30mg bid (n = 6)	AEB071 400mg bid and MEK162 30mg bid (n = 8)	
**Total**	**0**	**2 (33.3)**	**3 (60.0)**	**3 (50.0)**	**1 (16.7)**	**2 (50.0)**	**11 (33.3)**
Anemia	0	0	0	1 (16.7)	0	0	1 (3.0)
Diarrhea	0	0	2 (40.0)	1 (16.7)	0	0	3 (9.1)
Vomiting	0	0	0	1 (16.7)	1 (16.7)	1 (25.0)	3 (9.1)
Nausea	0	0	0	1 (16.7)	0	1 (25.0)	2 (6.1)
Fatigue	0	0	1 (20.0)	1 (16.7)	0	0	2 (6.1)
General physical health deterioration	0	0	1 (20.0)	0	0	0	1 (3.0)
Malaise	0	0	0	1 (16.7)	0	0	1 (3.0)
Increased blood creatinine	0	0	0	0	1 (16.7)	0	1 (3.0)
Decreased ejection fraction	0	0	0	0	0	1 (25.0)	1 (3.0)
Dermatitis acneiform	0	2 (33.3)	0	0	0	0	2 (6.1)
Rash	0	0	0	1 (16.7)	0	0	1 (3.0)

AEB071, sotrastaurin; bid, twice a day; MEK162, binimetinib.

Bold values summarizes the number of patients that were affected by any of the listed side effects.

Eleven deaths occurred during the study with the primary cause of death being disease progression. One non-treatment related death occurred due to gastrointestinal hemorrhage in a patient treated on dose level 3 (sotrastaurin 300 mg b.i.d. and binimetinib 30 mg b.i.d.).

### 3.3 Determination of the maximum tolerated dose

Eleven patients experienced DLTs across dose levels 2 through 6 (**Table 4A**). DLTs were mainly gastrointestinal in nature, including serious adverse events of nausea, vomiting, and diarrhea (**Table 4B**). The posterior distribution of DLT rates identified two dose combinations satisfying the EWOC criteria for the MTD: (1) sotrastaurin 300 mg b.i.d. and binimetinib 30 mg b.i.d.; and, (2) sotrastaurin 200 mg b.i.d. and binimetinib 45 mg b.i.d.

**Table 4A:** Plasma pharmacokinetic parameters for sotrastaurin by dose level for Cycle 1 Day 1 and Cycle 1 Day 15.

	Dose Level 1	Dose Level 2	Dose Level 3	Dose Level 4	Dose Level 5	Dose Level 6
	AEB071 150 mg bidandMEK162 45 mg bid (n = 6)	AEB071 200 mg bid andMEK162 45 mg bid(n = 5)	AEB071 300 mg bid andMEK162 30 mg bid(n = 6)	AEB071 300 mg bid andMEK162 45 mg bid(n = 6)	AEB071 350 mg bid andMEK162 30 mg bid(n = 6)	AEB071 400 mg bid andMEK162 30 mg bid(n = 8)
Cycle 1 Day 1 (C1D1):						
Number of patients with non-missing values	6	5	6	6	4	8
Median AUC_0–8hr_ (hr*ng/ml) **[Min; Max]**	8534.2 [3432.8; 12576.4]	7328.7 [5319.3; 9589.5]	16506.9 [6825.2; 27874.6]	14267.7 [7004.0; 23801.7]	20396.0 [11489.0; 26365.0]	17852.1 [5114.3; 39628.6]
Median C_max_ (ng/ml) [Min; Max]	2000.0 [970.0; 2530.0]	2070.0 [1300.0; 2810.0]	3300.0 [1250.0; 5280.0]	3210.0 [1570.0; 4110.0]	4540.0 [3410.0; 5800.0]	4370.0 [1670.0; 7020.0]
Median T_max_ (hr) [Min; Max]	1.6 [0.4; 4.0]	1.1 [1.0; 4.0]	1.5 [0.5; 2.0]	1.0 [0.5; 1.9]	1.0 [1.0; 2.0]	2.1 [0.5; 5.8]
Cycle 1 Day 1 (C1D1):						
Number of patients with non-missing values	6	4	2	5	5	5
Median AUC_0–8hr_ (hr*ng/ml) **[Min; Max]**	6068.0 [3665.0; 8328.4]	6320.8 [4775.8; 8696.8]	16864.4 [14796.3; 18932.5]	17571.6 [3977.1; 41857.3]	19990.7 [4367.4; 22336.3]	21648.8 [16899.9; 22542.5]
Median C_max_ (ng/ml) [Min; Max]	1255.0 [843.0; 1740.0]	1405.0 [965.0; 1770.0]	3305.0 [2070.0; 4540.0]	3950.0 [864.0; 7250.0]	3890.0 [1360.0; 6040.0]	4090.0 [2650.0; 4850.0]
Median T_max_ (hr) [Min; Max]	2.0 [1.1; 8.3]	1.5 [0.5; 2.0]	2.6 [1.0; 4.2]	3.9 [2.0; 4.2]	1.9 [0.5; 2.1]	2.1 [2.0; 8.0]
Median R_ACC_ [Min; Max]	0.7 [0.4; 1.8]	1.0 [0.5; 1.0]	1.0 [1.0; 1.1]	1.2 [0.6; 2.1]	1.0 [0.4; 1.1]	1.1 [0.6; 2.3]

AEB071, sotrastaurin; AUC_0–8hr_, AUC, area under the curve; AUC from time 0 to 8 h; bid, twice a day; C_max_, maximum plasma concentration; MEK162, binimetinib; PAS, pharmacokinetic analysis set; R_ACC_, accumulation ratio; T_max_, time point of maximum concentration.

The * symbol is used to denote multiplication in the formula hr*ng/ml.

**Table 4B:** Plasma pharmacokinetic parameters for binimetinib by dose level for Cycle 1 Day 1 and Cycle 1 Day 15.

	Dose Level 1	Dose Level 2	Dose Level 3	Dose Level 4	Dose Level 5	Dose Level 6
	AEB071 150 mg bidandMEK162 45 mg bid (n = 6)	AEB071 200 mg bid andMEK162 45 mg bid(n = 5)	AEB071 300 mg bid andMEK162 30 mg bid(n = 6)	AEB071 300 mg bid andMEK162 45 mg bid(n = 6)	AEB071 350 mg bid andMEK162 30 mg bid(n = 6)	AEB071 400 mg bid andMEK162 30 mg bid(n = 8)
Cycle 1 Day 1 (C1D1):						
Number of patients with non-missing values	6	5	6	6	5	8
Median AUC_0–8hr_ (hr*ng/ml) **[Min; Max]**	1542.8 [711.7; 3537.3]	1548.8 [1043.7; 2211.8]	1128.4 [511.4; 2178.5]	1613.7 [803.3; 2610.1]	1264.8 [457.2; 2124.1]	985.4 [540.8; 1937.5]
Median C_max_ (ng/ml) [Min; Max]	366.0 [202.0; 769.0]	450.0 [229.0; 761.0]	213.0 [120.0; 497.0]	370.0 [136.0; 569.0]	259.0 [141.0; 415.0]	279.0 [77.6; 388.0]
Median T_max_ (hr) [Min; Max]	1.1 [1.0; 3.8]	1.1 [1.0; 4.1]	2.0 [1.0; 2.1]	4.0 [0.5; 4.1]	2.0 [0.6; 2.3]	1.1 [0.5; 4.0]
Cycle 1 Day 1 (C1D1):						
Number of patients with non-missing values	6	4	4	4	5	4
Median AUC_0–8hr_ (hr*ng/ml) **[Min; Max]**	1995.9 [1015.3; 3041.6]	2137.7 [1121.6; 2772.3]	1213.3 [824.3; 3154.3]	1482.8 [1013.3; 2186.7]	1246.3 [532.0; 2641.4]	1449.4 [746.3; 1709.0]
Median C_max_ (ng/ml) [Min; Max]	493.0 [223.0; 748.0]	409.5 [299.0; 631.0]	246.0 [162.0; 917.0]	391.0 [195.0; 642.0]	280.0 [146.0; 865.0]	346.5 [136.0; 399.0]
Median T_max_ (hr) [Min; Max]	2.0 [1.1; 8.3]	1.6 [1.1; 4.0]	3.0 [1.0; 8.2]	2.9 [2.0; 4.1]	1.9 [0.5; 2.1]	1.5 [0.5; 8.0]
Median R_ACC_ [Min; Max]	1.3 [0.6; 1.6]	1.5 [1.1; 1.6]	0.8 [0.7; 2.5]	0.8 [0.6; 0.9]	1.4** [1.2; 2.3]	1.1 [0.5; 1.5]

**Number of patients with non-missing values was 4.

AEB071, sotrastaurin; AUC_0–8hr_, AUC, area under the curve; AUC from time 0 to 8 h; bid, twice a day; C_max_, maximum plasma concentration; MEK162, binimetinib; PAS, pharmacokinetic analysis set; R_ACC_, accumulation ratio; T_max_, time point of maximum concentration.

The * symbol is used to denote multiplication in the formula hr*ng/ml.

### 3.4 Efficacy

**Figure 1:**
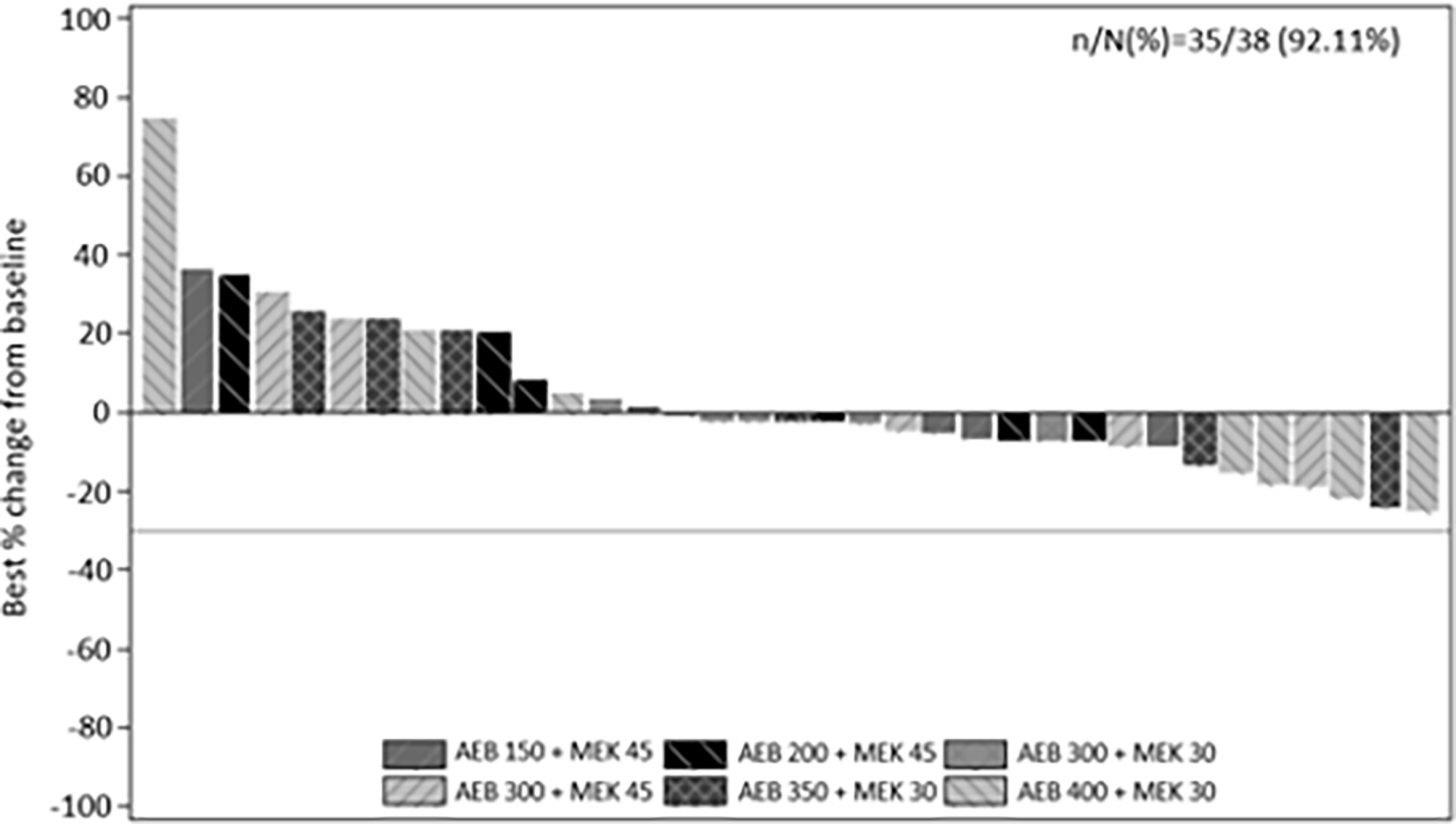
Best percentage change from baseline in sum of longest diameters as per investigator using RECIST v1.1. by treatment.

Thirty-five of the 38 treated patients were evaluable for radiographic response. Three patients had no post-baseline measurements. Stable disease was the best overall response in 23 patients (**Figure 1**). No complete or partial response per RECIST v1.1 criteria was observed. However, true tumor reduction was observed as evidenced by clinical images in one patient (**Supplementary Figure 1**) who had an unusual metastatic pattern with massive subcutaneous layers of tumors in addition to a diffuse metastatic disease to the liver.

The median progression free survival (PFS) based on the Kaplan-Meier method was 3.7 weeks (95% CI: 2.4 weeks, 3.8 weeks). The estimated PFS rate was 30.9% (95% CI: 15.3%, 46.4%) at 4 months, 20.6% (95% CI: 6.5%, 34.6%) at 6 months, 9.1% (95% CI: 0.0%, 20.1%) at 9 months, and 4.6% (95% CI: 0.0%, 12.9%) at 12 months.

### 3.5 Pharmacokinetic analysis

The exposure of sotrastaurin, as measured by median AUC_0-8hr_, in the presence of different doses of binimetinib, increased with increasing dose of sotrastaurin during cycle 1 day 1 as well as cycle 1 day 15. When the dose of sotrastaurin was increased from 150 mg to 400 mg b.i.d., the median AUC_0-8hr_ of sotrastaurin during cycle 1 day 15 increased from 6068.03 ng*hr/ml to 21648.83 ng*hr/ml (**Table 4A**). There was no observed accumulation of sotrastaurin following b.i.d. dosing when the exposure on day 15 was compared with that of day 1, as the median R_ACC_ ranged from 0.72 to 1.15 at different doses of sotrastaurin.

The exposure of binimetinib, as measured by median AUC_0-8hr_, increased when the dose was increased from 30 mg b.i.d. (median AUC_0-8hr_ range during cycle 1 day 15: 1213.33 to 1449.38 ng*hr/ml) to 45 mg b.i.d. (median AUC_0-8hr_ range during cycle 1 day 15: 1482.83 to 2137.70 ng*hr/ml) in the presence of varying doses (150 mg to 400 mg b.i.d.) of sotrastaurin, indicating increasing sotrastaurin dose levels had no significant effect on the PK of binimetinib (**Table 4B**). The accumulation potential of binimetinib following b.i.d. dosing in combination with sotrastaurin was difficult to assess in this study because the median R_ACC_ of three dose levels of binimetinib was much higher than 1 (1.34, 1.42, and 1.54) though the median R_ACC_ of the other three dose levels of binimetinib was lower than or close to 1 (0.76, 0.78, and 1.08).

These observed exposure values on cycle 1 day 15 of 45 mg binimetinib in combination with different doses of sotrastaurin in this study were consistent with values seen following administration of 45 mg binimetinib as single agent in the dose-expansion phase of the ARRAY-162-111 study in KRAS- and BRAF-mutant colorectal cancer patients (C_max_ ranged from 358 to 463 ng/mL and AUC_0-8hr_ ranged from 851 to 2310 hr*ng/mL).

The exposure values on C1D15 of 400 mg b.i.d. dose of sotrastaurin in combination with 30 mg binimetinib in this study were also consistent with the exposure values on C1D8 of 400 mg sotrastaurin b.i.d. in uveal melanoma patients in the COEB071X2102 study (C_max_ ranged from 2660 to 7390 ng/mL and AUC_0-8hr_ ranged from 13300 to 34400 ng*hr/mL).

### 3.6 Pharmacodynamic analysis

**Figure 2:**
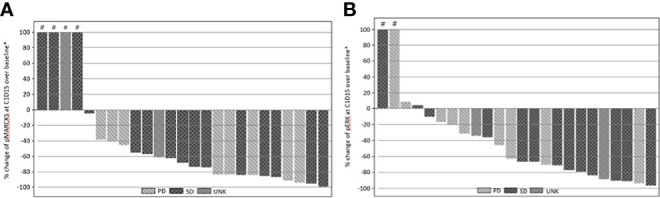
**(A)** Percentage change from baseline of pMARCKS at cycle 1 day 15 by best RECIST 1.1 overall response. **(B)** Percentage change from baseline of pERK at cycle 1 day 15 by best RECIST 1.1 overall response. Percentage change from baseline is greater than 100.

Inhibition of pMARCKS and pERK was observed in 21 of 25 cases, confirming effective PKC and MEK pathway inhibition. Inhibition of PKC as measured by reduction in normalized pMARCKS signal ranged from ~4% to ~99%, with 10 cases demonstrating a reduction in pMARCKS of 80% or more (**Figure 2A**). Inhibition of MEK as measured by a reduction in pERK ranged from ~10% to ~96%, with 6 cases demonstrating a reduction in pERK of 80% or more (**Figure 2B**). As no patients achieved a significant radiographic response, correlation of magnitude of pathway inhibition with efficacy is limited. Nine of 13 (69%) patients with suppression of pERK of 60% or greater achieved stable disease versus 4 of 11 (36%) patients with lower degrees of suppression, suggesting a potential association of more complete MAPK pathway inhibition and clinical efficacy.

## 4 Discussion

In this phase Ib clinical trial, we demonstrate for the first time the feasibility of concurrent inhibition of PKC and MEK in patients with advanced uveal melanoma; however, limited clinical activity was observed in this challenging patient population. Two dose combinations satisfying criteria for the maximum tolerated dose were identified: (1) sotrastaurin 300 mg and binimetinib 30 mg; and, (2) sotrastaurin 200 mg and binimetinib 45 mg. Exposure to both drugs in combination was consistent with single-agent data for either drug, indicating no PK interaction between sotrastaurin and binimetinib.

While either dosing combination may have been declared the recommended phase 2 dose, the study investigators determined that further clinical and pharmacodynamic data were required to make this decision. In the setting of MAPK inhibition with vemurafenib in patients with advanced BRAF mutant melanoma, greater than 80% inhibition of ERK phosphorylation correlated with clinical response (21). A similar direct relationship between target inhibition and response has been previously reported with MEK inhibition in uveal melanoma (14). Although the majority of treated patients on this study achieved some degree of target inhibition, the depth of target inhibition varied widely across patients, with only 10 of 25 cases demonstrating a reduction in pMARCKS of 80% or more and 6 of 25 cases demonstrating a reduction in pERK or 80% of more, suggesting the need to optimize dosing such that more complete target inhibition is achieved. When revisiting the preclinical studies assessing concurrent PKC/MEK inhibition, only doses that achieved complete pERK inhibition consistently suppressed cell growth (18). *In vivo* studies had used a cell line with an EIF1AX mutation which is typically found in non-metastasizing tumors. Future preclinical studies should include cell lines derived from metastatic specimens or representing those genomic subgroups most commonly found in patients, such as cells harboring BAP1 alterations.

In all patients treated across the 6 dose levels tested, stable disease was achieved in 60.5% with an estimated PFS rate at 12 months of 4.6%. These findings are similar to those of a literature review of 40 studies involving systemic treatment for metastatic uveal melanoma, where the mean ORR was 4.6% and stable disease was observed in 30.8% of patients (22). The PFS ranged from 1.8 - 7.1 months in studies where PFS was reported. Interestingly, in the current study, 69% of patients with suppression of pERK of 60% or greater achieved stable disease versus 36% of patients with lower degrees of suppression, supporting a potential association of more complete MAPK pathway inhibition and clinical efficacy.

Treatment associated toxicity was commonly observed across all dose levels tested, with 97.4% of patients experiencing a treatment related adverse event and 68.4% of patients experiencing an adverse event of grade 3-4 in severity. Gastrointestinal toxicity, including severe, cisplatin-like nausea, was particularly challenging for patients with responded only moderately to anti-emetic treatments. Although there have been reports of central serous retinopathy associated with binimetinib and other MEK and PKC inhibitors (23), in our study only 5.3% (2/38) of patients experienced retinopathy regardless of causality and no grade 3 or 4 events were observed. Nevertheless, the overall toxicity burden of this treatment regimen limited drug exposure that can be achieved in patients and may impact treatment efficacy.

Based upon the challenges with toxicity and dosing, as well as the limited efficacy signals observed, the phase II portion of this phase Ib/II trial was not initiated and the development of this particular drug combination for uveal melanoma discontinued; however, the therapeutic strategy of optimizing PKC inhibition in advanced uveal melanoma alone or in combination with other agents remains of great interest. Preclinical data supports the further clinical evaluation of PKC inhibition alone or in combination with other inhibitors of MEK, MET or FAK (24), and efficacy may be achieved with next generation inhibitors of PKC with improved toxicity profiles. Daravosertib (IDE916; LXS196) is a next generation selective PKC inhibitor targeting both the novel and classical PKC isoforms with a toxicity profile distinct from sotrastaurin. A prior study of this agent administered alone demonstrated encouraging clinical activity with a manageable toxicity profile (25), and it is currently being studied alone and in combination with either binimetinib or crizotinib in patients with advanced uveal melanoma (NCT03947385). This study will provide further insights into the therapeutic role of PKC inhibition in this disease.

## Data availability statement

The raw data supporting the conclusions of this article will be made available by the authors, without undue reservation.

## Ethics statement

The studies involving human participants were reviewed and approved by The Institutional Review Boards and EthicsCommittees of each participating institution. The patients/ participants provided their written informed consent toparticipate in this study. Written informed consent was obtained from the individual(s) for the publication of any identifiable images or data included in this article.

## Author contributions

All authors listed have made a substantial, direct, and intellectual contribution to the work and approved it for publication.

## References

[1] Jager MJ Shields CL Cebulla CM Abdel-Rahman MH Grossniklaus HE Stern M-H. Uveal melanoma. Nat Rev Dis Primers (2020) 6:24. doi: 10.1038/s41572-020-0158-0 32273508

[2] Singh AD Turell ME Topham AK. Uveal melanoma: trends in incidence, treatment, and survival. Ophthalmology (2011) 118:1881–5. doi: 10.1016/j.ophtha.2011.01.040 21704381

[3] Kujala E Makitie T Kivela T. Very long-term prognosis of patients with malignant uveal melanoma. Invest Ophthalmol Vis Sci (2003) 44:4651–9. doi: 10.1167/iovs.03-0538 14578381

[4] Khoja L Atenafu EG Suciu S Leyvraz S Sato T Marshall E. Meta-analysis in metastatic uveal melanoma to determine progression free and overall survival benchmarks: An international rare cancers initiative (IRCI) ocular melanoma study. Ann Oncol (2019) 30:1370–80. doi: 10.1093/annonc/mdz176 31150059

[5] Rantala ES Hernberg M Kivela TT. Overall survival after treatment for metastatic uveal melanoma: a systematic review and meta-analysis. Melanoma Res (2019) 29:561–8. doi: 10.1097/CMR.0000000000000575 PMC6887637 30664106

[6] Nathan P Hassel JC Rutkowski P Baurain J-F Butler MO Schlaak M. Overall survival benefit with tebentafusp in metastatic uveal melanoma. N Engl J Med (2021) 385:1196–206. doi: 10.1056/NEJMoa2103485 34551229

[7] Carvajal RD Nathan P Sacco JJ Orloff M Hernandez-Aya LF Yang J. Phase I study of safety, tolerability, and efficacy of tebentafusp using a step-up dosing regimen and expansion in patients with metastatic uveal melanoma. J Clin Oncol (2022). doi: 10.1200/JCO.21.01805 PMC9177239 35254876

[8] Van Raamsdonk CD Bezrookove V Green G Bauer J Gaugler L O'Brien JM. Frequent somatic mutations of GNAQ in uveal melanoma and blue naevi. Nature (2009) 457:599–602. doi: 10.1038/nature07586 19078957 PMC2696133

[9] Van Raamsdonk CD Griewank KG Crosby MB Garrido MC Vemula S Wiesner T. Mutations in GNA11 in uveal melanoma. N Engl J Med (2010) 363:2191–9. doi: 10.1056/NEJMoa1000584 PMC3107972 21083380

[10] Johansson P Aoude LG Wadt K Glasson WJ Warrier SK Hewitt AW. Deep sequencing of uveal melanoma identifies a recurrent mutation in PLCB4. Oncotarget (2016) 7:4624–31. doi: 10.18632/oncotarget.6614 PMC4826231 26683228

[11] Moore AR Ceraudo E Sher JJ Guan Y Shoushtari AN Chang MT. Recurrent activating mutations of G-protein-coupled receptor CYSLTR2 in uveal melanoma. Nat Genet (2016) 48:675–80. doi: 10.1038/ng.3549 PMC5032652 27089179

[12] Decatur CL Ong E Garg N Anbunathan H Bowcock AM Field MG. Driver mutations in uveal melanoma: Associations with gene expression profile and patient outcomes. JAMA Ophthalmol (2016) 134:728–33. doi: 10.1001/jamaophthalmol.2016.0903 PMC4966162 27123562

[13] Piperno-Neumann S Larkin J Carvajal RD Luke JJ Schwartz GK Hodi FS. Genomic profiling of metastatic uveal melanoma and clinical results of a phase I study of the protein kinase c inhibitor AEB071. Mol Cancer Ther (2020) 19:1031–9. doi: 10.1158/1535-7163.MCT-19-0098 32029634

[14] Carvajal RD Sosman JA Quevedo JF Milhem MM Joshua AM Kudchadkar RR. Effect of selumetinib vs chemotherapy on progression-free survival in uveal melanoma: a randomized clinical trial. JAMA (2014) 311:2397–405. doi: 10.1001/jama.2014.6096 PMC4249701 24938562

[15] Bendell JC Javle M Bekaii-Saab TS Finn RS Wainberg ZA Laheru DA. A phase 1 dose-escalation and expansion study of binimetinib (MEK162), a potent and selective oral MEK1/2 inhibitor. Br J Cancer (2017) 116:575–83. doi: 10.1038/bjc.2017.10 PMC5344293 28152546

[16] Dummer R Ascierto PA Gogas HJ Arance A Mandala M Liszkay G. Overall survival in patients with BRAF-mutant melanoma receiving encorafenib plus binimetinib versus vemurafenib or encorafenib (COLUMBUS): a multicentre, open-label, randomised, phase 3 trial. Lancet Oncol (2018) 19:1315–27. doi: 10.1016/S1470-2045(18)30497-2 30219628

[17] Evenou JP Wagner J Zenke G Brinkmann V Wagner K Kovarik J. The potent protein kinase c-selective inhibitor AEB071 (sotrastaurin) represents a new class of immunosuppressive agents affecting early T-cell activation. J Pharmacol Exp Ther (2009) 330:792–801. doi: 10.1124/jpet.109.153205 19491325

[18] Chen X Wu Q Tan L Porter D Jager MJ Emery C. Combined PKC and MEK inhibition in uveal melanoma with GNAQ and GNA11 mutations. Oncogene (2014) 33:4724–34. doi: 10.1038/onc.2013.418 PMC4524511 24141786

[19] Li C Takahashi C Zhang L Huseni M Stankovich B Mashhedi H. Development of a robust flow cytometry-based pharmacodynamic assay to detect phospho-protein signals for phosphatidylinositol 3-kinase inhibitors in multiple myeloma. J Transl Med (2013) 11:76. doi: 10.1186/1479-5876-11-76 23522020 PMC3623880

[20] Tighiouart M Liu Y Rogatko A. Escalation with overdose control using time to toxicity for cancer phase I clinical trials. PLoS One (2014) 9:e93070. doi: 10.1371/journal.pone.0093070 24663812 PMC3963973

[21] Bollag G Hirth P Tsai J Zhang J Ibrahim PN Cho H. Clinical efficacy of a RAF inhibitor needs broad target blockade in BRAF-mutant melanoma. Nature (2010) 467:596–9. doi: 10.1038/nature09454 PMC2948082 20823850

[22] Buder K Gesierich A Gelbrich G Goebeler M. Systemic treatment of metastatic uveal melanoma: review of literature and future perspectives. Cancer Med (2013) 2:674–86. doi: 10.1002/cam4.133 PMC3892799 24403233

[23] van Dijk EH van Herpen CM Marinkovic M Haanen JBAG Amundson D Luyten GPM. Serous retinopathy associated with mitogen-activated protein kinase kinase inhibition (Binimetinib) for metastatic cutaneous and uveal melanoma. Ophthalmology (2015) 122:1907–16. doi: 10.1016/j.ophtha.2015.05.027 26123090

[24] Ma J Weng L Bastian BC Chen X. Functional characterization of uveal melanoma oncogenes. Oncogene (2021) 40:806–20. doi: 10.1038/s41388-020-01569-5 PMC7856047 33262460

[25] Kapiteijn E Carlino M Boni V Loirat D Speetjens F Park J. Abstract CT068: A phase I trial of LXS196, a novel PKC inhibitor for metastatic uveal melanoma. Cancer Res (2019) 79:CT068–CT. doi: 10.1158/1538-7445.AM2019-CT068 PMC10006169 36624219

