# Supplementary figures and images for: A phase Ib trial of combined PKC and MEK inhibition with sotrastaurin and binimetinib in patients with metastatic uveal melanoma
Source: Front Oncol. 2023 Jun 9;12:975642. doi: 10.3389/fonc.2022.975642 (PMC10288853; doi:10.3389/fonc.2022.975642)

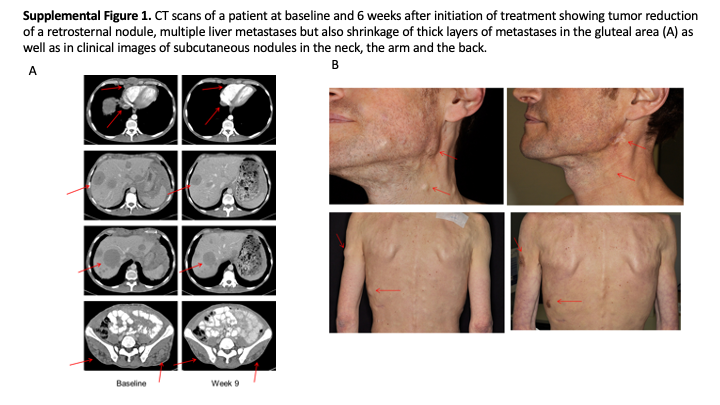

Supplement: Supplementary file 1 [file Image_1.tiff]
