# Supplementary material for: A phase Ib trial of combined PKC and MEK inhibition with sotrastaurin and binimetinib in patients with metastatic uveal melanoma
Source: Front Oncol. 2023 Jun 9;12:975642. doi: 10.3389/fonc.2022.975642 (PMC10288853; doi:10.3389/fonc.2022.975642)
Supplement: Supplementary file 2 [file Table_1.docx]

|  | Dose Level 1 | Dose Level 2 | Dose Level 3 | Dose Level 4 | Dose Level 5 | Dose Level 6 | All  patients (n = 38) |
| --- | --- | --- | --- | --- | --- | --- | --- |
|  | AEB071 150 mg bid  and  MEK162 45 mg bid  (n = 6) | AEB071 200 mg bid and MEK162 45 mg bid  (n = 6) | AEB071 300 mg bid and  MEK162 30 mg bid  (n = 6) | AEB071 300 mg bid and  MEK162 45 mg bid  (n = 6) | AEB071 350 mg bid and  MEK162 30 mg bid  (n = 6) | AEB071 400 mg bid and MEK162 30 mg bid  (n = 8) |  |
| All deaths | 4 (66.7) | 1 (16.7) | 1 (16.7) | 3 (50.0) | 1 (16.7) | 1 (12.5) | 11 (28.9) |
| On-treatment deaths | 3 (50.0) | 1 (16.7) | 1 (16.7) | 1 (16.7) | 1 (16.7) | 1 (12.5) | 8 (21.1) |
| Adverse events | 6 (100) | 6 (100) | 6 (100) | 6 (100) | 6 (100) | 8 (100) | 38 (100) |
| Serious adverse events | 3 (50.0) | 1 (16.7) | 3 (50.0) | 4 (66.7) | 2 (33.3) | 6 (75.0) | 19 (50.0) |
| Adverse events leading to discontinuation | 0 | 2 (33.3) | 1 (16.7) | 0 | 0 | 1 (12.5) | 4 (10.5) |
| Adverse events requiring dose interruption and/or change | 4 (66.7) | 5 (83.3) | 5 (83.3) | 5 (83.3) | 4 (66.7) | 7 (87.5) | 30 (78.9) |
|  |  |  |  |  |  |  |  |

**Supplemental table 1.** Deaths, serious adverse events, and significant adverse events.

AEB071, sotrastaurin; bid, twice a day; MEK162, binimetinib.
